# High-throughput fecundity measurements in *Drosophila*

**DOI:** 10.1038/s41598-018-22777-w

**Published:** 2018-03-13

**Authors:** Pierre Nouhaud, François Mallard, Rodolphe Poupardin, Neda Barghi, Christian Schlötterer

**Affiliations:** 1 Institut für Populationsgenetik, Vetmeduni, Vienna, Austria; 2 Present Address: UMR 8197 IBENS, Paris, France

## Abstract

Fecundity is probably the most frequently studied fitness component in *Drosophila*. Nevertheless, currently used methods to measure fecundity are not well-suited for large-scale experiments, with many populations being assayed in parallel. Here we present a standardized pipeline to measure fecundity in many *Drosophila* population samples with substantially reduced hand on times. Using a high-contrast medium for egg laying, we developed a Java plug-in for ImageJ to quantify the number of eggs by image processing. We show that our method is fast and provides reliable egg counts.

## Introduction

Over the past years, *Drosophila* has been a widely used model in evolutionary biology because of its ease-of-use and short generation time. Of central interest has been the characterization of life history traits (e.g., life span, fecundity, mating competitiveness) under laboratory conditions as they are closely linked to fitness and are thus predicted to be under strong directional selection^1^. Female fecundity, estimated by the number of eggs laid, is a widely used proxy for fitness^2^.

Because manual egg counting is labor intensive, several methods have been developed for automated egg counting, all relying on image analysis. Quantifly, a java tool based on machine learning, has been designed for counting eggs automatically from images^3^. This method is attractive as it needs little manipulation, but it is mainly designed for small surfaces (i.e. narrow vials), as the software only processes low resolution pictures (≤756 × 756 pixels). In another approach^4^ eggs are laid on hard agar, washed and transferred on a black filter paper using a vacuum filtration system through a large Büchner funnel. A picture of the eggs on black paper is taken with a desktop scanner and analyzed using the particle count plug-in of the ImageJ software^5^. This method is reliable and precise but the substantial hands-on time limits its use for large-scale experiments. Other imaging methods have been developed for amphibian or mosquito models but are not adapted for large-scale experiments in *Drosophila*^6,7^.

Automatic egg counting using imaging methods faces many challenges: eggs can be unevenly distributed, making clumps or having variable orientations and shapes. Moreover, when pictures are directly taken from the medium on which flies lay eggs, heterogeneity of the medium and rough surfaces can result in false positives. Another important factor is the softness of the medium as it affects the number of eggs laid and is highly dependent on the assayed species^8^.

Fecundity is a highly plastic trait, strongly affected by population density^9–15^. For a reliable comparison of fecundity data, it is essential to minimize the influence of environmental factors by maintenance in a common environment for a number of generations, i.e. common garden. Density in common garden experiments (CGE) is best controlled by counting the number of eggs. Again, manual counting is highly time-consuming, precluding large-scale experiments.

In this study, we developed a standardized pipeline to measure fecundity in *Drosophila* at the population level. We first developed a density control procedure to transfer a specific amount of eggs into bottles containing *Drosophila* medium in order to rear different populations under a CGE. To reduce transgenerational effects, we typically control density for at least two generations. We quantify the number of eggs on a dedicated medium by coupling a camera to a dedicated image processing pipeline. The whole procedure was designed to minimize the manipulation of flies and avoid time-consuming steps.

## Methods

### Egg density control

Manual egg counting is a tedious and time-consuming task. We developed a simple yet fast and efficient method to transfer quickly and precisely a user-defined number of eggs into bottles/vials using a pipetting procedure based on a protocol developed by Clancy & Kennington^16^. The protocol detailed below is designed for setting up 5 bottles each containing 400 eggs. If different number of bottles and eggs are desired, the protocol can be easily adjusted. In the evening, adult *Drosophila simulans* flies are transferred into embryonic collection cages (Cat. #59–101, Genesee Scientific, San Diego, California) with a Petri dish containing a dedicated egg-laying medium (4% agar and 4% sucrose topped with 1 ml yeast paste). The yeast paste stimulates egg laying^17–19^ and the high agar concentration prevents inserting eggs in the agar. Egg laying should be kept as short as possible to prevent hatching of eggs (note the temperature dependence of developmental rates). Eggs laid on the surface are easily collected on the next day using a fine brush. Eggs are placed on a fine net and yeast paste is removed by careful rinsing with tap water (at ambient temperature). Eggs are transferred into a 1.5 ml Eppendorf tube containing 200 µl PBS (1 mM Calcium chloride dihydrate, 0.5 mM Magnesium Chloride Hexahydrate, pH = 7.4). After settling of eggs at the bottom of the tube, the volume of eggs is compared to a range of tubes containing 50 to 70 μl of PBS by increments of 5 μl. The volume of eggs is then adjusted to 60 µl through visual comparisons by pipetting excess eggs with a clipped tip. Excess PBS is removed from the tube and 1200 µl PBS (i.e., 20x the initial egg volume) is added to the eggs. They are suspended by gently blowing them in and out of a clipped tip (to reduce sheering forces that could damage the eggs). 200 µl of this suspension is transferred to a bottle containing standard *Drosophila* medium and contains around 400 eggs (see Results).

With the size and volume of eggs varying between species/populations, this protocol requires a prior calibration to adjust the volume of the suspension. We validated our method by manually counting the number of eggs after pipetting under a stereomicroscope. The deviation between the observed and expected number of eggs (400) was tested using a Wilcoxon test.

### Egg hatchability assay

The procedure described above is dedicated to efficiently transfer a precise number of eggs to into new food medium. Because the larvae density rather than the egg density is critical in experiments, we quantified the incidence on the egg hatchability of our two successive manipulations (rinsing the eggs with tap water and their immersion in PBS). We collected freshly laid eggs (<6 hours) from a *D*. *simulans* population split into nine embryonic collection cages with a Petri dish containing a medium that stimulates egg-laying (37.5 gr agar, 1.5 L water, 50 gr sucrose, 500 ml grape juice). The eggs collected from each cage were randomly assigned to one of the three following treatments: (i) eggs were washed with tap water and immersed in PBS for 15 minutes; (ii) eggs were washed with tap water only; (iii) eggs were not washed at all. For each cage, three independent sets of 50 eggs were manually counted and transferred individually with a brush to fresh grape plates. After 40 hours, the number of eggs hatched to larvae was counted within each plate.

To test for the effect of washing the eggs with water and PBS we fitted a generalized linear mixed model (using a binomial distribution) with one fixed categorical effect (treatment) and 3 levels (described above). Replicates of each cage were treated as random effects. Significance of the fixed effect was tested using ANOVA F-tests.

### Preparation of a high-contrast medium for fecundity assays

Proper quantification of the number of eggs by image analysis requires a dark medium against which the light eggs provide a strong contrast. Furthermore, the medium needs the right softness - in too soft medium eggs will not remain on the surface and too hard medium results in reduced egg laying. Throughout this study, we used ROTH Agar-Agar, Kobe 1 (density 0.55). Note that different agar types can affect the softness of the final media and thus the flies egg-laying.

To determine the proper softness of the medium, we tested the fecundity on media with different agar concentrations at 0.7, 1, 2 and 3%. Populations of 100 *D*. *simulans* flies each laid eggs during 36 hours in population cages. For each concentration, we measured the number of eggs for two replicated populations. Every 12 hours the high-contrast medium plates were replaced with new ones to avoid egg hatching (i.e., a “transfer”), and eggs were manually counted under a stereomicroscope. We tested for the effect of the recipe on egg counts using a generalized linear mixed model (setting agar concentration as a fixed effect and population and transfer as random effects) followed by a *post-hoc* Tukey’s honest significant difference (HSD) test with correction for multiple testing.

We obtained best results with this recipe: 950 ml water, 6.9 g agar, 5 g active charcoal and 19 g yeast yield one liter medium, resulting in an agar concentration of 0.7%. The medium is autoclaved (20 min at 120 °C), and when cooled down to 65–70 °C, 83 g malt syrup and 1.3 g Nipagin diluted in 4.2 ml 96% EtOH are added. After stirring, the medium is poured into Petri dishes and bubbles are removed using a Bunsen burner flame and sterile pipette tips.

### Picture acquisition

Pictures need to be in focus, thus constant framing and lighting are key for high-quality data. We used a Canon EOS 750D with a 18–55 mm lens (Canon, Tokyo, Japan) held in a fixed position, 30 cm above the high-contrast medium plate using a wall mount bracket (see Fig. S1 for the camera setup). The specific DLSR camera model does not matter as long as a sufficiently high resolution (>15 millions pixels), a full manual mode and the possibility of remote control by a computer are provided. We used guides below the camera to facilitate the orientation of the Petri dish relative to the camera - thus allowing for rapid change of Petri dishes. The background behind Petri dishes was white for high contrast to the dark agar in the Petri dishes. The light source was a SCHOTT KL 1600 LED system covered with a paper to diffuse the light and avoid strong reflections.

Pictures were taken using a 2-second exposure, F16 aperture, and 200 iso (high iso values will increase the noise; parameters may change depending on the light and camera setup). The camera was remotely controlled by a computer using EOS Utility 2 software (Canon, Tokyo, Japan). Focus was done manually (the use of a small aperture - F16 - facilitates manual focusing) using the “Live View” function of the software. Using the automatic software timer further facilitates a rapid data acquisition.

To provide reliable results, it is critical that all parameter values (e.g., light source, zoom value, white balance, picture resolution, aperture) remain constant throughout the course of the experiment.

### Quantification of the number of eggs with ImageJ

We developed a Java plug-in for ImageJ software^5^ to perform batch analyses of pictures (File S1). The plug-in automatically detects the area of interest (dark and circular area on a white background) using a wand tracing tool. Based on the particle detection program of ImageJ, it first transforms colored pictures into 8-bit grey pictures and then into simple black and white pictures using different thresholds of detection (Fig. S2). Particles within the area of interest (i.e., the plate) are detected along with their position on the picture (xy coordinates), surface area (in pixels^2^), perimeter, the lengths of their major and minor dimensions (in pixels) and the angle between these two dimensions.

This plug-in is derived from the “Batch population sensor” described in Mallard *et al*.^20^ (see File S1 of the original paper for a complete description). It is designed to analyze recursively multiple images automatically. This plug-in was initially designed to analyze a set of pictures containing moving particles on a fixed background. It was modified here to analyze pictures individually but most of the initial settings and options have been kept. It includes multiple image pre-treatments, the possibility to scale the pictures using a visual mark, and several options that allow an efficient recognition of the area of interest (here the Petri dish boundaries). The default options have been optimized for our settings but are fully described in the original paper. Here, we included an additional option that restricts the analysis to a given file extension, that is not available in the initial version. It is set by default to”.JPG” in the first option pop-up window after the input and output directories selection and can be inactivated.

For each picture, all metrics are saved in a spreadsheet named according to the directory path of the picture used as input file (Fig. S3). Combined with a suitable directory structure, this feature allows storing any suitable information that could be useful for follow up analyses (e.g., population ID, transfer, treatment).

In addition to eggs, the plug-in also detects particles present on the high-contrast medium (e.g., dust). Therefore, we developed an R script to derive empirical filtering steps. The main function combines the tables exported by the ImageJ plug-in for each picture and filters eggs assuming a bimodal distribution of the particles area, with the first mode corresponding to dust and other small particles and the second mode to eggs (Fig. S4).

### Validation of the pipeline for fecundity estimates

We tested whether the automated egg counting provides accurate numbers by comparing the obtained numbers with manual egg counts in a fecundity assay with two *D*. *simulans* populations. After two generations of density control under common garden conditions, five replicates of 100 individuals were transferred into large embryo collection cages for each population. Five transfers were made and for each the number of eggs was estimated by our pipeline and manually counted under a stereomicroscope. The concordance between manual counts and the automated estimates was determined by Spearman’s rank correlation.

Unless stated otherwise, all data analysis and statistical tests were performed in R version 3.3.2^21^.

### Data availability

The R script developed for statistical analyses and data visualization is available in Dataset 1. All data generated during the current study (including raw pictures), the Java plug-in for ImageJ and its associated R script for post-processing are available on FigShare (https://figshare.com/projects/High-throughput_fecundity_measurements_in_Drosophila/27562).

## Results and Discussion

### Reliable transfer of defined egg quantities by pipetting

**Figure 1:**
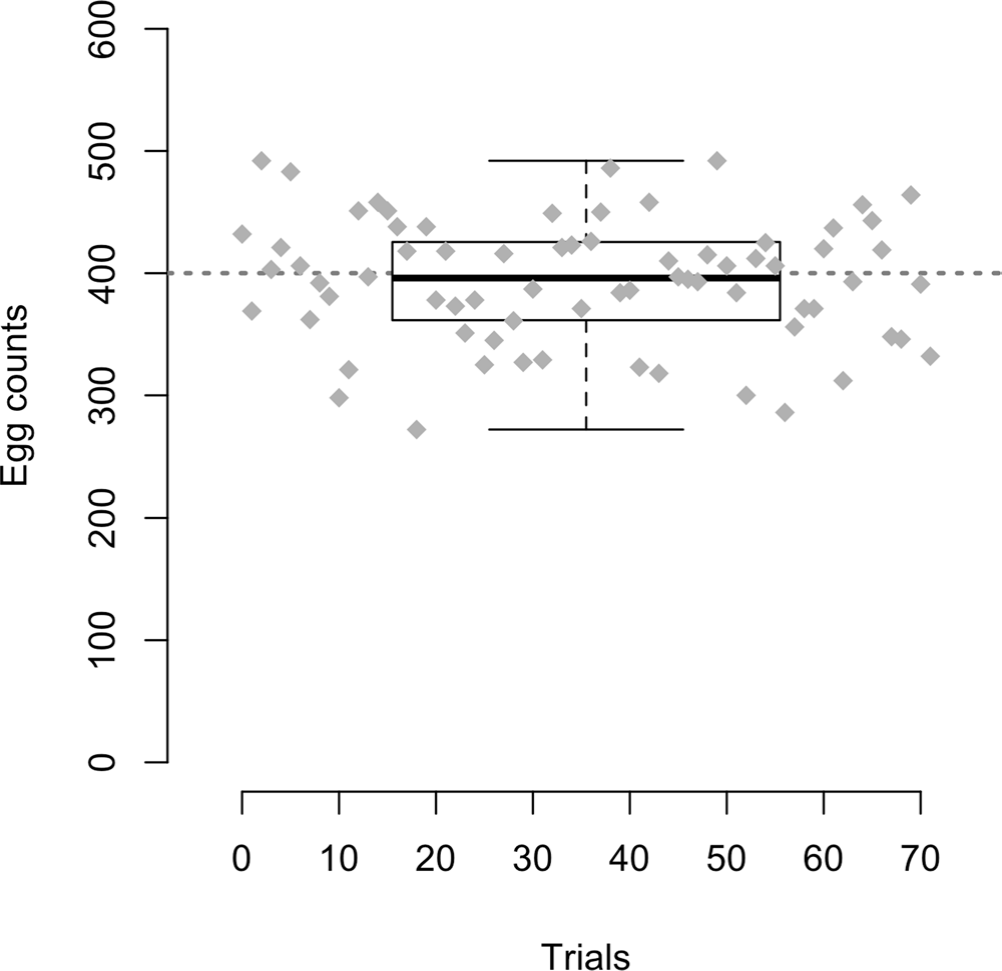
Pipetting reliably transfers the targeted number of eggs. 72 pipetting trials, each aiming for 400 eggs were performed. Eggs were counted manually under a stereomicroscope. The box plot summarizes the entire experiment, while the gray diamonds indicate the egg counts for each trial.

We tested the accuracy of the pipetting method by counting manually 72 trials, each with 200 µl suspension containing about 400 eggs (Fig. 1). On average, we counted 394 eggs (sd = 50.9), with half of the measurements comprised between 371 and 434, showing that our pipetting protocol allows for a controlled distribution of 400 eggs (Wilcoxon test, *P* = 0.94). It is a fast and accurate approach to control for egg density in large-scale experiments, with five bottles being set up in less than five minutes.

### Washing eggs does not affect hatchability

To rule out that our procedure affects the final larvae density in the medium, we tested whether our protocol affects the mean egg hatchability. We found no significant difference between the number of hatched eggs that were 1) not washed at all (40.89 ± 2.15 out of 50), 2) washed with water only (42.38 ± 2.77) and 3) washed with water and transferred to PBS (41.56 ± 2.46, generalized linear model, *P* = 0.94).

### Softness of medium affects fecundity

**Figure 2:**
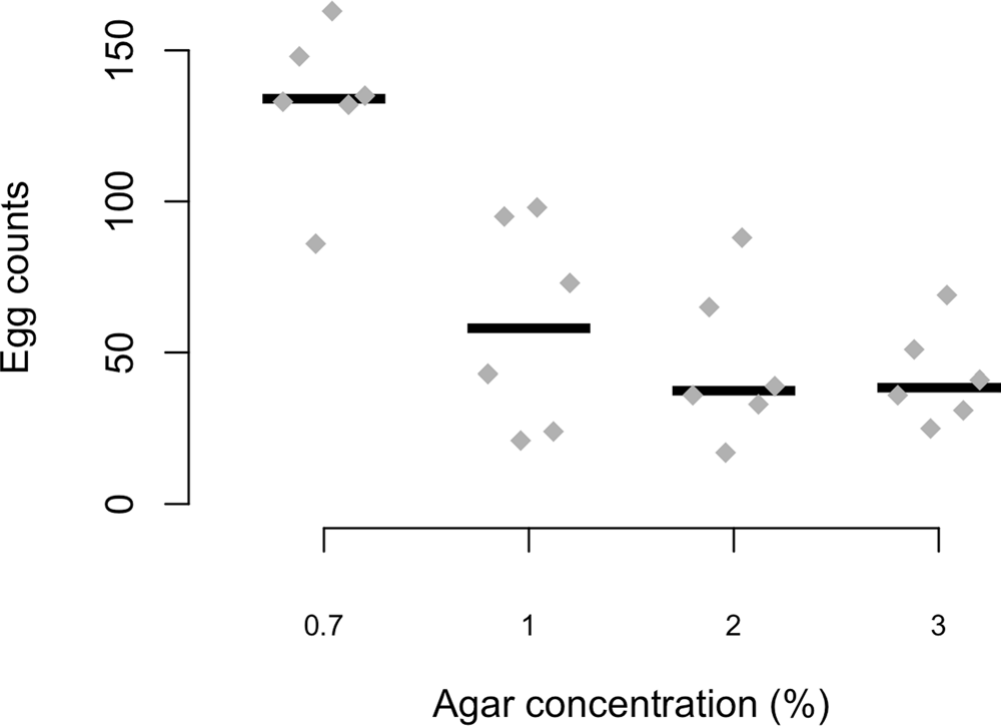
Effect of agar concentration on the number of eggs laid. At each concentration, six replicated populations (grey diamonds) of 100 *D*. *simulans* each laying eggs for 12 hours and eggs were counted manually. Black bars depict medians. No significant difference in egg counts was detected between recipes at 2% and 3%, all other comparisons were significantly different at *P* < 0.05 (GLMM followed by Tukey’s HSD test, see main text for *P*-values).

We evaluated the impact of the agar concentration on the number of eggs laid. We notice a marked influence of the agar concentration on the number of eggs laid – higher agar concentrations resulted in fewer eggs (Fig. 2 and Data S2, generalized linear mixed model followed by Tukey’s HSD test with correction for multiple testing, *P*_0.7–1%_ < 0.001, *P*_0.7–2%_ < 0.001, *P*_0.7–3%_ < 0.001, *P*_1–2%_ = 0.013, *P*_1–3%_ < 0.001, *P*_2–3%_ = 0.69). A difference of 0.3% agar reduces the number of eggs by 50% (Fig. 2, median_0.7%_ = 133, median_1%_ = 59). Based on these results, we used an agar concentration of 0.7% for the rest of the experiments, which also matches the agar concentration in our standard *Drosophila* medium.

### Reliable egg counts with the automated procedure

**Figure 3:**
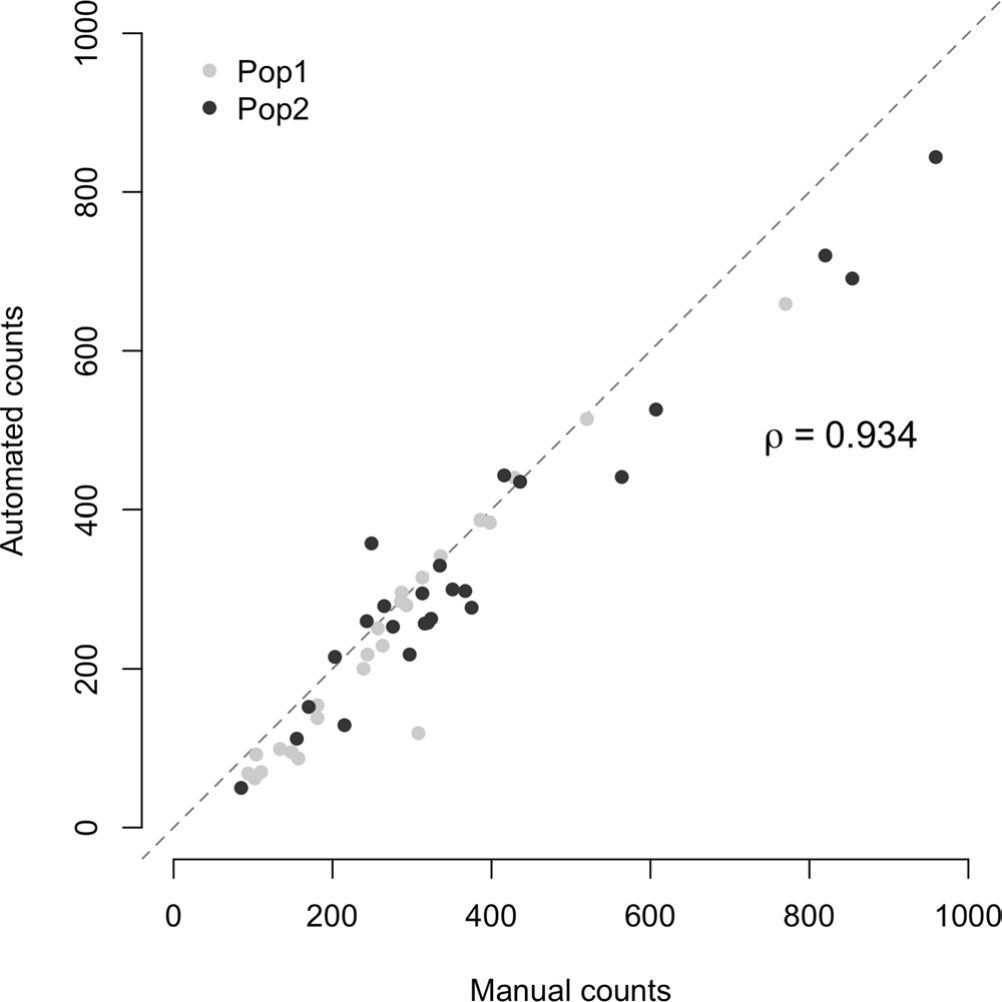
Accuracy of the automated egg counting procedure. The number of eggs was measured for two populations of 100 *D*. *simulans* individuals for which fresh high-contrast medium plates were provided every 12 hours (i.e. a “transfer”). After each transfer, eggs were counted manually and automatically. Each population was replicated five times, and five transfers were made. The automated procedure provides reliable results similar to the manual counting (Spearman’s rank correlation, ρ = 0.934). The dashed line has a slope of one.

To ensure that our computer-assisted method provides reliable egg counts, we subjected the same set of high-contrast medium plates to both manual and automated counting procedures. The two methods provided very similar results (Spearman’s rank correlation, ρ = 0.934, Fig. 3). However, at very high egg densities (>500), the automated estimates are downward biased. Visual inspection of the plates revealed that this is due to egg clustering on the medium. Our automated procedure considers egg clusters as a single big particle that is filtered out because of its size, hence underestimating egg counts. Decreasing the number of flies assayed in each population cage avoids this bias of the automated procedure.

## Conclusion

Here, we present an integrated pipeline to control for egg density and measure fecundity in *Drosophila* laboratory experiments. Designed to avoid time-consuming steps while maintaining a high level of precision, our method is particularly suited for large-scale experiments where many populations are assayed in parallel.

While this protocol has been extensively tested in *D*. *simulans*, it would require minor adjustments depending on the species and experimental parameters of interest (e.g., volume to be pipetted for density control, hardness of the medium, population census size for the fecundity assay).

## Electronic supplementary material

Dataset 1

Supplementary information
