# Supplementary material for: High-throughput fecundity measurements in Drosophila
Source: Sci Rep. 2018 Mar 13;8:4469. doi: 10.1038/s41598-018-22777-w (PMC5849729; doi:10.1038/s41598-018-22777-w)
Supplement: Supplementary file 2 — Supplementary information [file 41598_2018_22777_MOESM2_ESM.docx]

**High-throughput fecundity measurements in *Drosophila***

**Supplementary information**

**Authors**

Pierre Nouhaud^1,*^, François Mallard^1,2,*^, Rodolphe Poupardin^1,*^, Neda Barghi^1^, Christian Schlötterer^1^

**Affiliations**

^1^: Institut für Populationsgenetik, Vetmeduni, Vienna, Austria

^2^: Current address: UMR 8197 IBENS, Paris, France

**Corresponding author:**

Christian Schlötterer^1^, christian.schloetterer@vetmeduni.ac.at

* These authors contributed equally to this work.

**
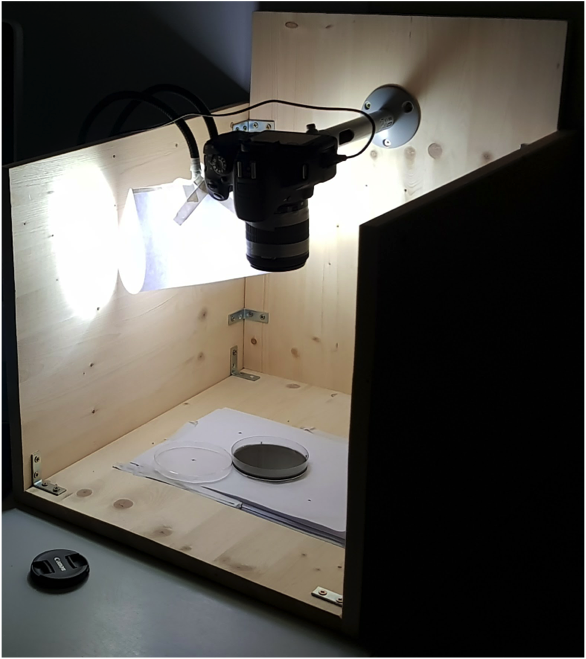
Figure S1. Experimental setup used for the acquisition of pictures.** A DSLR remotely controlled by a computer is attached to a wall bracket stand. Lighting is provided by a LED system with a diffuser to provide a homogenous light.

**
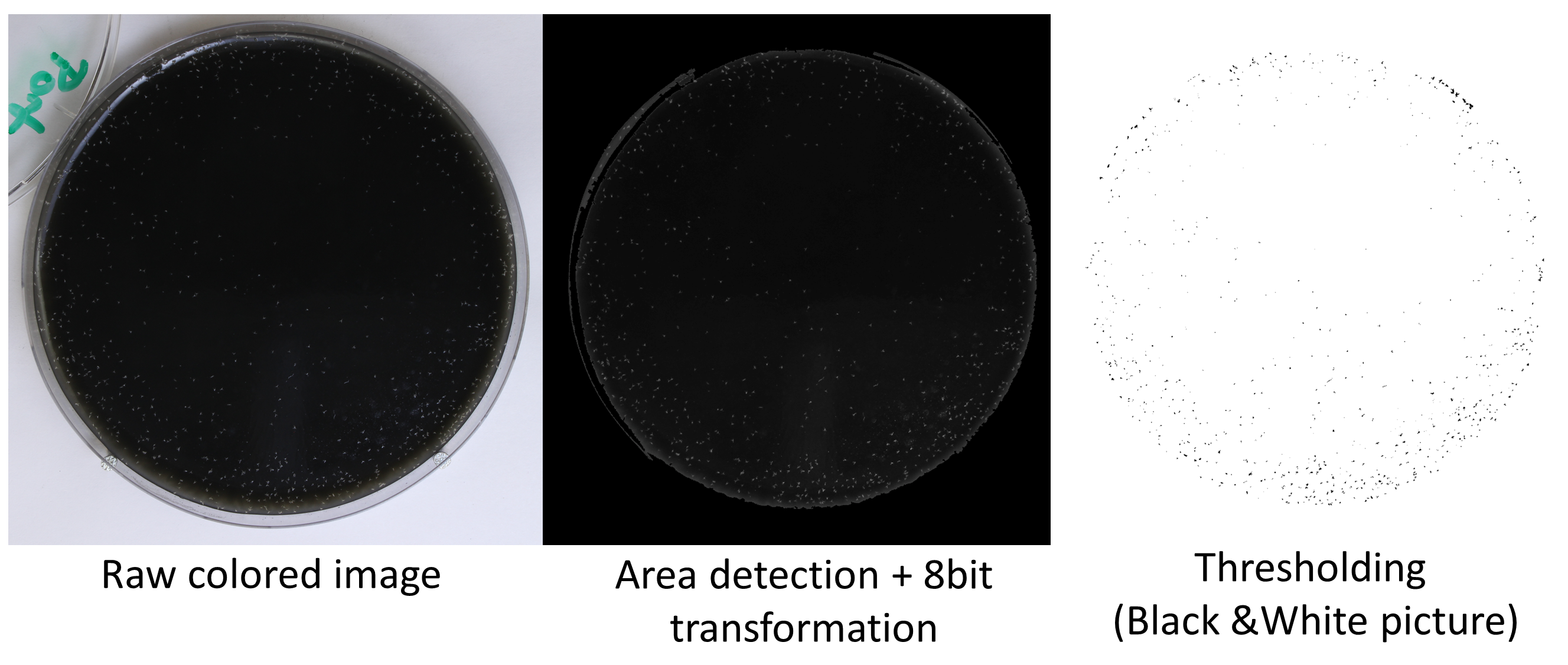
Figure S2. Image processing approach.** RAW pictures are automatically cropped to the area of interest (using Wand tool) and converted into 8-bit grey pictures. Then, a thresholding approach is applied to distinguish between eggs (black) and the background (white) using a custom Java plug-in for ImageJ.


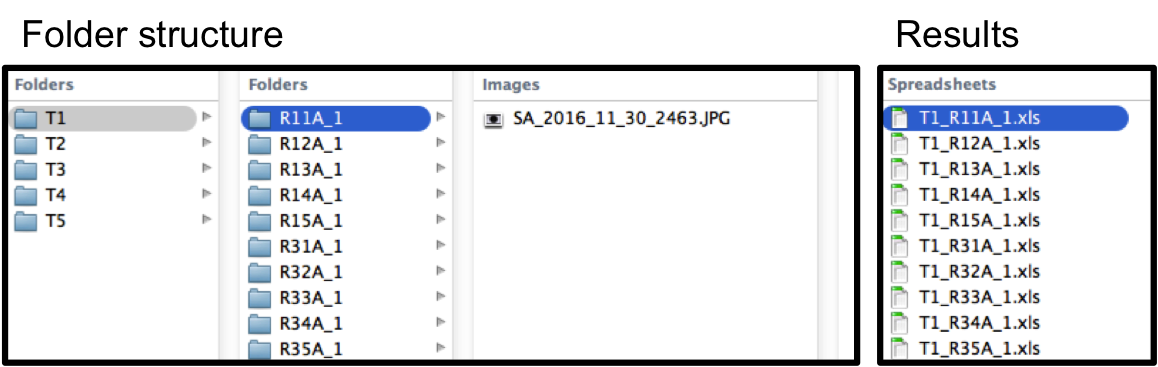


**Figure S3. Example of the folder structure containing pictures (left) and associated output of the Java plug-in for ImageJ (right).** The results files provided by the Java plug-in are named according to the directory path in which pictures are located.


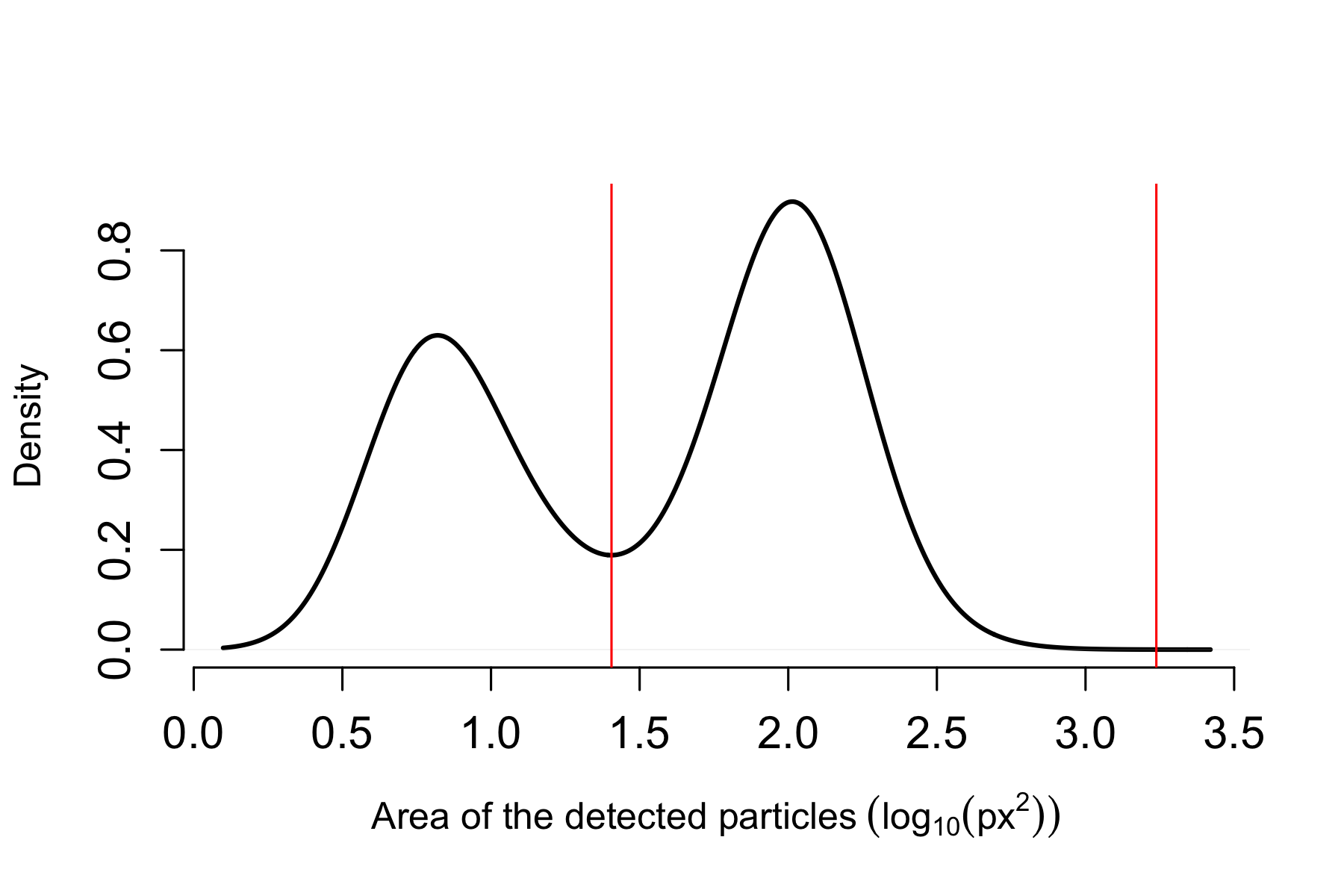
**Figure S4. Filtering of egg particles.** Using a custom R function, we divide the area distribution of the detected particles (in log_10_(pixels^2^)) to keep only the eggs. Red vertical lines show the cutoffs automatically detected and applied to our data set.
